# A Lexical Approach to Identifying Dimensions of Organizational Culture

**DOI:** 10.3389/fpsyg.2018.00876

**Published:** 2018-06-05

**Authors:** Derek S. Chapman, Paige Reeves, Michelle Chapin

**Affiliations:** Department of Psychology, University of Calgary, Calgary, AB, Canada

**Keywords:** lexical, organizational culture, job attitudes, corporate climate, culture measurement

## Abstract

A comprehensive measure of organizational culture was developed using a lexical approach, a method typically employed within the study of personality. 1761 adjectives were narrowed down and factor analyzed, which resulted in the identification of a nine factor solution to organizational culture, including the dimensions of: Innovative, Dominant, Pace, Friendly, Prestigious, Trendy, Corporate Social Responsibility, Traditional, and Diverse. Comprised of 135 adjectives most frequently used in describing organizational culture by current employees of several hundred organizations, the Lexical Organizational Culture Scale (LOCS) was found to predict employee commitment, job satisfaction, job search behaviors, and subjective fit better than earlier scales of organizational culture.

## Introduction

Since the 1980’s, nearly 5000 studies have been conducted on organizational culture, which showcases its importance within the literature (Hartnell et al., 2011). Defined as “the set of shared, taken-for-granted implicit assumptions that a group holds and that determines how it perceives, thinks about and reacts to its various environments,” (Schein, 1996, p. 236), organizational culture can be thought of as the collection of values, beliefs, and assumptions which influence employee attitudes and behaviors (Schein, 2004). Understanding the shared values and beliefs among employees has been shown to predict a number of key individual and organizational outcomes. While an exhaustive review of the full culture/employee outcome literature is beyond the scope of this article, we suggest the reader consult several meta-analytic reviews demonstrating the complex relationships among many culture variables and individual employee outcomes (e.g., Harter et al., 2002; Parker et al., 2003). As Parker et al. (2003) note, the confusion around the conceptualization and measurement of organizational culture/climate has made definitive conclusions about the relationships among culture variables and individual employee outcomes difficult. We report some individual study results next to illustrate the variety of scholarly work being conducted in this area. For example, certain organizational cultures have been found to positively relate to employee attitudes such as organizational commitment, subjective fit perceptions, and job satisfaction, positive organizational outcomes such as objective organizational profit and growth, and other individual outcomes such as turnover (O’Reilly et al., 1991; Sheridan, 1992; Johnson and McIntye, 1998; Hartnell et al., 2011). In addition, Johnson and McIntye (1998) found that among current employees, job satisfaction was significantly related to subjective factors such as perceived organizational creativity and innovation. Many other researchers have found significant correlations between a wide variety of organizational culture variables and employee attitudes such as job satisfaction. For example, McKinnon et al. (2003) found positive relationships between cultural measures of innovation, respect for people, aggressiveness, and stability with job satisfaction. In an Australian study, Lok and Crawford (2004) found that employees who reported higher scores on innovative and supportive cultures also reported higher job satisfaction. Others have found negative associations between hierarchical cultures and job satisfaction (e.g., Goodman et al., 2001; Lund, 2003). Our purpose is not to revisit whether culture/climate factors are important for individual employee outcomes (they are) but rather, to attempt to determine and measure culture factors with a comprehensive lexical approach in an effort to capture the most meaningful employee conceptualizations of organizational culture/climate and ultimately to improve our prediction of these outcomes.

Past research on organizational attraction has found that both objective factors (e.g., pay, location) and subjective factors (e.g., perceptions of innovativeness or being traditional) influence whether or not a person will pursue job opportunities with a particular organization (Lievens and Highhouse, 2003; Slaughter et al., 2004; Chapman et al., 2005; Lievens, 2007; Lievens et al., 2007).

This effect extends past consumers and potential applicants. Given the strong implications these subjective factors have for job applicants and job incumbents alike, it is necessary to determine a complete set of organizational dimensions present in the conscious of potential applicants and employees, and thereby discover the extent to which these dimensions influence applicant and employee behavior.

There have been numerous approaches used to define and describe organizational culture that have enriched our understanding of this important concept. A recent review of the climate and culture literatures argues that there may be a benefit to revisiting our conceptualization of climate and culture into an overall global conception or ‘gestalt’ of the organization (Schneider et al., 2011, 2017). However, we believe there are limitations of existing methods that warrant exploring novel approaches to identify a taxonomy of corporate culture. For example, the lexical approach that proved so beneficial for identifying taxonomies of human personality such as the Five Factor Model (FFM) (see Goldberg, 1993 for a review) and the HEXACO model (Lee and Ashton, 2004) has not been used to examine the construct of organizational culture. Accordingly, the primary aim of this study is to identify the factor structure of organizational culture from the ground up, using a comprehensive lexical strategy similar to that employed by personality researchers.

Another major limitation of earlier measures of culture is a small number of company targets being assessed. Often only a few very large companies (e.g., IBM for Hofstede’s work) have been used to identify the culture taxonomy (Hofstede, 1980). We believe this poses an issue with respect to deficiencies in defining the factor space due to a lack of variability. For example, if only large and successful Fortune 500 companies are described, the taxonomy is unlikely to include variables such as size and success of the company. Thus, a second aim of this study is to examine corporate culture using a very large number of target companies of all sizes and industries.

The third aim of our study is to compare our lexically derived structure to several existing measures of culture to determine if the lexical approach and broader target approaches provides a taxonomy with better prediction of meaningful organizational outcomes beyond what is already captured by existing frameworks.

### Historical Approaches

We will begin by describing some of the many innovative approaches researchers and practitioners have used in the past to define and describe organizational culture. A full description of all existing culture measures is beyond the scope of this study. An excellent review can be found at Jung et al. (2009). Instead we will highlight the approaches typically used and attempt to highlight some of the strengths and limitations of these approaches.

#### Qualitative

Researchers have been interested for some time in determining the best way to describe organizational culture. Many practitioners and organizational researchers believe that culture is too complex and rich to be captured by quantitative survey methods (Alvesson, 2013). From this perspective, it is unlikely that surveys would capture the right type of data to identify the idiosyncrasies of an organization’s culture. This position is somewhat analogous to the classic ‘broken leg’ argument from the decision making literature whereby quantitative statistical approaches are criticized for being inadequate for capturing the complexities of decision making (Meehl, 1954). Specifically, a statistical algorithm to predict whether a person will watch a particular movie would fail to capture the fact that a particular subject has a broken leg and therefore be inaccurate.

While qualitative work has made significant contributions to our understanding of culture, there are some considerable limitations associated with describing companies qualitatively. For example, it has been argued that because individual practitioners/researchers carry their individual biases with them into the assessment process, it is unlikely that two researchers describing a company’s culture independently would arrive at the same description (Dutton et al., 1994). Furthermore, qualitative methods, while providing richness and flexibility, are time consuming and costly (Mays and Pope, 1995) deterring their use by medium and small sized companies that could benefit most from managing their culture. In addition, as critics of the broken leg argument would assert, qualitative researchers are prone to overestimate the influence of company idiosyncrasies, on, in this case, employee attitudes (Alvesson, 2013). Lastly, qualitative approaches make it difficult or impossible to compare cultures from one company to another, creating a barrier to understanding culture across organizations and forcing researchers to rely on case studies of individual companies (Schein, 1990). This makes it difficult to generalize findings beyond a particular organization.

#### Strategy Based

Strategy scholars have also been keenly interested in corporate culture. After all, corporate culture is seen as a key lever to use in implementing business strategies (Crittenden and Crittenden, 2008). As a result, many top down strategic approaches to describing corporate culture have been developed. For example, the Competing Values Framework places companies in one of four quadrants (e.g., Adhocracy, Clan, Market, or Hierarchy; Cameron and Quinn, 1999). While this provides us with a useful taxonomy that has been used successfully in research, these approaches are limited in several important ways. First, the taxonomy is top down. Strategic approaches start with the strategy and then impose that structure on the companies, whereas bottom up approaches use employee perceptions as the basis of the taxonomy. The key advantage of employee-based taxonomies is they are better able to capture the dimensions that are meaningful to employees when they think about their companies. In other words, whereas strategy based measures may be highly effective at assessing the extent to which employees are aligned with a particular strategy, they may be less useful in predicting other important cultural outcomes such as employee attitudes and behaviors.

Second, it is likely that these simplified strategic taxonomies are too limited to capture the complexity of organizational culture. For example, it is unlikely that there are only four types of cultures. Thus a broader measure should be more effective at describing the company and predicting outcomes.

#### Deductive Approach

Historically, researchers have assessed subjective culture of organizations by using instruments derived from brand personality and human personality literature, for example the Symbolic Trait Dimensions created by Lievens et al. (2005). This methodology is potentially problematic in that it may inadvertently exclude words that are specific to the organizational context, as neither brand personality nor human personality studies were originally intended to capture the essence of organizational culture. As such, the subsequent dimensions elicited may not adequately capture organizational culture.

A review of the literature revealed that the instrumental/symbolic framework used extensively in marketing research has been useful in capturing the dimensions along which individuals evaluate organizational image and culture. According to Lievens and Highhouse (2003), instrumental factors are objective, factual attributes of a job/organization (e.g., pay, location), and are a major determinant of organizational attraction. Symbolic attributes “convey symbolic company information in the form of imagery and general trait inferences that applicants assign to organizations” (Lievens et al., 2007, S48). Past research has found that symbolic attributes ascribed to an organization differentiate between organizations in the same industry to a greater extent than objective factors (Highhouse et al., 1999) and add incremental variance over and above instrumental attributes in attracting potential applicants to organizations (Lievens and Highhouse, 2003; Slaughter et al., 2004; Lievens et al., 2005; Lievens, 2007). A recent study found that symbolic trait inferences explained a significant proportion of variance in organizational attractiveness over and above objective factors for potential applicants, actual applicants, and employees for the Belgian Army (Lievens, 2007). Lievens et al. (2007, S55) argued that “if organizations only focus on instrumental job and organizational attributes (as is traditionally the case), an important part of what makes an organization an attractive employer is ignored.”

Lievens and Highhouse (2003) applied the instrumental/symbolic framework to examine organizational image by soliciting the ratings of the traditional job attributes and subjective traits of five Belgian banks. Results showed that trait inferences, conceived as a bank’s personality, add incremental variance over and above job/organizational attributes in the prediction of a company’s attractiveness as a place to work. Furthermore, given that organizations within the same industry often offer similar pay and benefits, the finding that it was also easier to differentiate among the banks on the basis of trait inferences than traditional job attributes is important. The traits used in this study to assess symbolic facets are limited by the fact that items were drawn from Aaker’s (1997) Brand Personality Scale, which was derived from descriptive traits of people rather than organizations. In a pre-study, the researchers asked 20 participants to rate each of the 42 items on the extent to which it was descriptive of a bank’s ‘personality’, which ended with 23 trait adjectives that loaded onto five dimensions (Sincerity, Innovativeness, Competence, Prestige, and Robustness). However, the list of adjectives examined is specific to the banking industry, making it unclear if they can be generalized to organizational image or culture as a whole.

Expanding on this research, Lievens et al. (2005) examined factors that influence organizational attractiveness in the Belgian army among potential applicants. The authors developed a measure based on Cable and Turban’s (2001) dimensions of employer attractiveness, in which trait inferences are only one part of the overall theory, alongside job and organizational attributes and employer familiarity. Trait dimensions, again, were captured using Aaker’s (1997) Brand Personality Scale. They found that trait inferences contributed most variance, followed by job and organizational attributes. Prospective applicants were asked to rate each of Aaker’s adjectives on their suitability for describing the Belgian Army, which resulted in a final measure of 25 descriptive items incorporated into a six dimension symbolic scale (SbS) including the dimensions of Sincerity, Cheerfulness, Excitement, Competence, Prestige, and Ruggedness. The use of brand personality as a base to elicit organizational traits may not have provided a comprehensive adjective list for describing an organization, and once again the factors that emerged may be idiosyncratic to the army and not fully capture the dimensions upon which individuals view organizational image or culture in general.

Lievens et al. (2007) were not the only authors around this time to create measures of organizational image. Slaughter et al. (2004) conducted a series of studies to develop a measure of symbolic perceptions of organizational personality and to examine their relationship with organizational attraction. Results from factor analysis revealed five dimensions placed into a scale named the Organizational Personality Scale (OPS) labeled: Boy Scout (indicative of a friendly, cooperative organization), Innovativeness (unique, creative), Dominance (successful, popular), Thrift (low class, poor), and Style (hip, trendy). We believe that this approach is extremely helpful for understanding organizational image and culture; however, a few limitations warrant discussion.

One major issue that must be raised with this study is the possibility of range restriction in the sampling of the company domain. The organizations used to establish dimensions of organizational personality in this study are very similar in important respects. JC Penny, Disney, and Wal-Mart, for example, are all very large corporations, known to virtually all participants (as must be the case in a study using real world organizations with non-employees), and as such may not capture dimensions more salient to small, local companies for which many of the participants may themselves work. This is also problematic because words that are descriptive and unique to higher end retailers, (e.g., Saks Fifth Avenue, Bloomingdales), professional organizations (e.g., law firms, oil, and gas), or smaller less dominant organizations were excluded from consideration, but are crucial in establishing a taxonomy which can be generalized across companies and industries.

The image that individuals hold of an organization is based on the information available to them (Gatewood et al., 1993). Available information could be derived from a number of sources including (i) personal experience with a company’s products and services; (ii) interaction with employees of an organization; (iii) the experiences of friends and family; or (iv) multiple media sources such as advertising campaigns, corporate literature, and internet groups (Lemmink et al., 2003; Slaughter et al., 2004).

Some researchers have called for a more integrated approach to assessing organizational image and culture that incorporates both current employees and potential applicants (Lievens et al., 2007) and current employees and customers (Davies et al., 2004). Internal views of organizational culture have been captured in scales that are similar to scales developed to measure organizational personality. For example, O’Reilly et al. (1991) developed the Organizational Culture Profile (OCP) that has seven dimensions that contain items that potentially describe external views of an organization, i.e., words that reflect innovation and stability. Although potential applicants and current employees may have different perceptions of an organization (Lemmink et al., 2003), a comprehensive organizational trait taxonomy would encompass the major dimensions in which organizations vary and therefore would be a useful tool to evaluate different stakeholder perspectives. The development of a robust set of dimensions applicable to both applicants and job incumbents is important for researchers who wish to compare organizations both within similar and different industries. Unfortunately, the creation of the OCP, like the previous scales, was based on known human personality dimensions, rather than an inductive approach which could identify purely organizational descriptive characteristics.

Davies et al. (2004) developed the Corporate Character Scale (CCS) to assess both employee and customer views of organization reputation and, similar to Slaughter et al. (2004, p. 127) used the personification metaphor. The authors define corporate character as “how a stakeholder distinguishes an organization, expressed in terms of human characteristics.” To obtain a list of organizational descriptive words, the authors first established four dimensions based on a literature review from brand and human personality as well as corporate reputation studies and conducted extensive qualitative research to generate items for the initial dimensions. Ultimately, they established five major and two minor dimensions to the CCS labeled: Agreeableness (friendly, honest, and concerned), Enterprise (cool, imaginative, and daring), Competence (reliable and ambitious), Chic (charming, refined, and snobby), and Ruthlessness (arrogant and authoritarian). The personification metaphor used in this research and others mentioned is problematic for several reasons. It is possible that by having participants imagine that an organization ‘comes to life as a person’ that it delineates boundaries and primes interpretation of the words, thereby limiting the scope of representative organizational words and distorting the obtained factor structure that emerges. Essentially, organizations are *not* people and do not possess human qualities (Morgeson and Hofmann, 1999). While many consider this a useful metaphor in understanding organizational image and culture, is it necessary? The lexical strategy offers an alternative strategy to the question of how to garner a comprehensive list of potential organization-specific descriptive words in which to establish dimensions to evaluate organizational variation.

#### Lexical Approach

The lexical approach has provided the framework for compiling a large set of representative variables on which to establish dimensions of personality variation. It is a purely inductive approach, which has served as the basis for the Big Five factor structure of personality (see Goldberg, 1993, for review) and more recently the HEXACO model, or six factor model of personality (Lee and Ashton, 2004). In using a lexical approach, all possible descriptive words are considered, rather than any set of predetermined words. Goldberg (1982, p. 204) argued that fundamental to the lexical strategy is the assumption that “those individual differences that are most significant in daily transactions of persons with each other will eventually become encoded into their language.” Ashton et al. (2007, p. 1516) add that “based on this assumption, we would expect that any major axis of personality variation would be represented by many adjectives, each of which would describe some manifestation of the underlying dimension.” Given that organizational entities play a large role in individuals’ lives, it follows that those words that people use when describing organizations when talking with others would also become encoded in the language. Analogous with the concept of personality traits, organizational descriptive words would be those used by people to distinguish one organization from another and differentiate between the views of people about the same organization (Davies et al., 2004).

This method of research incorporates both the symbolic and instrumental approaches (words that describe subjective organizational trait inferences and objective factors alike), as both have been argued to be important to organizational attraction. The lexical approach also provides the opportunity to determine if various stakeholders (i.e., potential applicants, existing employees, customers etc.) hold similar concepts of organizational image and culture.

Although the lexical strategy is not without criticism, it offers an alternative approach to compiling a representative set of organizational descriptive words on which to conduct factor analysis. Ashton and Lee (2005, p. 7) argued that the lexical strategy has much strength as a basis for establishing dimensions of the personality construct:

The chief value of the lexical hypothesis is not merely that it allows the researchers to develop a long catalog of the personality attributes used by speakers of a given language. Instead, the primary significance of the lexical hypothesis is that it provides a strategy for research aimed at identifying the major dimensions of personality variation – that is, a relatively small set of roughly independent axes along which people differ in their typical behavioral tendencies.

A criticism of the lexical approach is the use of adjectives as variables is not necessarily representative of the complexity inherent in the construct of personality (Ashton and Lee, 2005). Although the lexical hypothesis does not imply the exclusive use of adjectives, adjectives do capture important differences in language and can be applied in varying degrees, yielding more utility when evaluating differences (Saucier and Goldberg, 1996). For example, one organization may be considered highly innovative whereas another may be somewhat innovative. Typically, these levels of difference cannot be caught when nouns are used as descriptors.

The primary goal of the present study focuses on determining the factor structure of organizational culture using a lexical strategy. As such, the first research question is proposed as follows:

R1: What is the factor structure of organizational culture when a lexical methodology is used?

A secondary purpose of this study is to investigate how the dimensions gleaned from the study interact with various outcome variables. Decades of research have found that organization culture and climate variables predict important organizational outcomes such as job satisfaction, person-organization fit, safety adherence, performance, and affective commitment to the organization (see Schneider et al., 2017 for a more detailed review). Accordingly, the first hypothesis of this study is:

H1: The LOCS will significantly predict four outcome variables: (a) job satisfaction (b) job search behaviors (c) subjective person-organization fit (d) affective commitment.

We expect that the important dimensions of organizational culture identified in this study will be more predictive of outcome measures than previous scales intended for the same use, including the Organizational Personality Scale (OPS) (O’Reilly et al., 1991), Corporate Character Scale (CCS) (Davies et al., 2004), and symbolic scales (SbS) (Lievens et al., 2005), due to the approach used in creating the scale. The lexical strategy enables us to identity dimensions that are specific to organizations due to the fact that we are not using preconceptions to guide variable selection. Basically, we start from ground zero, whereas other scales started with a preconceived variable set that may have excluded words and dimensions that are integral to organizational culture. Our methodology will develop a comprehensive, organization-specific, precise measure of organizational culture, thus allowing for more accurate predictions of the outcome variables we are investigating. Therefore, we make the following predictions:

H2: Lexically-derived culture dimensions add incremental predictive variance above and beyond the (a) OPS, (b) CCS, and the (c) SbS in the prediction of job satisfaction.
H3: Lexically-derived culture dimensions will add incremental predictive variance above and beyond the (a) OPS, (b) CCS, and the (c) SbS in the prediction of job search behaviors.
H4: Lexically-derived culture dimensions will add incremental predictive variance above and beyond the (a) OPS, (b) CCS, and the (c) SbS in the prediction of subjective person-organization fit.
H5: Lexically-derived culture dimensions will add incremental predictive variance above and beyond the (a) OPS, (b) CCS, and the (c) SbS in the prediction of affective commitment.

## Materials and Methods

### Pilot Study – Initial Instrument Development

The purpose of the pilot study was to refine the word list generated utilizing the lexical strategy. Following the lexical strategy, 1,761 potential organization descriptive terms, all adjectives, were extracted from the second edition of the Oxford Dictionary of English (Soanes and Stevenson, 2005). The criteria used for inclusion in the initial instrument were, (1) any adjective that could potentially be used to describe and/or evaluate an organization; and (2) archaic and rare words were excluded from consideration. The first author further reduced this list by identifying words that were deemed inappropriate to the context of organizational culture or were considered too difficult or obscure. Overall agreement between the authors on the first cut of the items was 96%. In an iterative process the first and second authors discussed the elimination of individual adjectives from the list. There was a 100% agreement on whether to retain or eliminate the remaining 71 items. The final compilation of 1,689 adjectives were randomized and divided into four word list each containing approximately 422 adjectives.

### Pilot Study Participants

Sixty university students from a large, Canadian institution participated in this pilot study (70% women, mean age 21.5 years). The students received course credits for their participation.

### Measures

#### Organizational Adjective List

As outlined above, participants received one of four word lists containing approximately 422 terms.

#### Demographics

This questionnaire contained basic demographic information including, age, cultural identity, education, employment status, hours of work per week, and items pertaining to work history.

### Procedure

Each participant was given a survey to complete, which consisted of the demographic questionnaire and one of four word lists containing approximately 422 adjectives. Participants were asked to read each word carefully and rate how often he or she used the word in conversation with others when describing attributes of any given organization (1 = *never*, 5 = *very frequently*).

Descriptive statistics were performed on the resulting data. In order to shorten the word lists, those words that received a mean rating of 2.66 or higher (the approximate midpoint) were reviewed by the researchers. Twenty-one of the remaining 458 words were eliminated because they were deemed extremely evaluative (e.g., horrible and fantastic) and not useful in describing organizational variation. The resulting and final word list contained 437 items, which were then used in the main study.

### Main Study – Determining Image Dimensions

#### Participants

Participation in the main study was contingent upon two conditions: (1) each participant must be employed either full-time or part-time at the time of their participation, and (2) each participant must have been working for their current employer for a minimum of 3 months. These conditions were chosen to ensure that all participants had the necessary time to consider one and only one company, and to develop a durable subjective perception of the company’s culture, as previous research has shown that differences exist in the perception of organizational image between those in the applicant stage and job incumbents (Lievens, 2007).

Three hundred and forty-three employed undergraduate students participated in the main study. Of these, 18 surveys were incomplete and the data collected from these participants removed from analysis (completion rate of 93.7%). In total, data from 325 participants was included in this study. Participants’ mean age was 21.01 years (*SD* = 3.51). The majority of subjects were women (75.7%) and the ethnic breakdown was as follows: 62.5% Caucasian, 22.5% Asian, 2.3% Middle Eastern, 2% Latin-American, 1.5% Black, and 7.6% Other. All students that registered for the study were assigned bonus credits in return for their participation.

The majority of participants were employed part-time (94.5%). Participants worked in a variety of occupational backgrounds including customer service (*n* = 83), sales (*n* = 57), administration (*n* = 27), hospitality (*n* = 24), health care (*n* = 17), management (*n* = 9), operations (*n* = 8), accounting (*n* = 6), human resources (*n* = 4), and research (*n* = 4).

#### Measures

##### Organizational descriptive variables

The 437 item word list developed in the pilot study was used as the measure for organizational descriptor items. Participants were asked, “To what extent do the following adjectives accurately describe the organization for whom you are presently employed?” and items were rated on a seven point Likert type scale ranging from 1 (to no extent) to 7 (to a very great extent).

##### Job satisfaction

The five-item Brayfield and Rothe (1951) job satisfaction scale was used to measure individuals’ level of satisfaction with their current job. Sample items include “I feel fairly satisfied with my present job” and “Most days I am enthusiastic about my work.” Items were rated on a seven point Likert scale, ranging from 1 (strongly disagree) to 7 (strongly agree).

##### Job search behaviors

Items adapted from Blau’s (1994) measure of active job search behavior were used to assess employees’ job search activities. This six-item subscale included questions measuring the extent that employees sent resumes to potential employers and filled out job applications. Participants were asked to indicate the frequency with which they carried out each of the behaviors within the last 6 months, and responses were based on a 5-point scale, ranging from 1 (never) to 5 (very frequently).

##### General subjective fit

A five-item measure of general subjective P-O fit was used. These items, which were adapted from Piasentin and Chapman (2007), included: “I fit in well with other people who work in my organization,” “Other people in my organization would say that I am a good fit with the company,” “I often feel like I am not well suited to the company I work for” (reverse keyed), “Overall, I feel that my organization is a good match for me,” and “I would probably fit in better at another organization than the one I currently work for” (reverse keyed).

##### Affective commitment

Allen and Meyer’s (1990) eight item affective commitment scale was used to measure the extent that participants were affectively committed to their organizations. Sample items include “This organization has a great deal of personal meaning for me” and “I do not feel ‘emotionally attached’ to this organization” (reverse keyed).

##### Previous measures of subjective organizational culture

Participants were also asked to rate the adjectives included in three previous subjective organizational culture scales: the Corporate Character Scale (CCS) which comprised 49 items in 6 factors (Davies et al., 2004), the Symbolic Trait Dimensions (SbS) which comprised a total of 18 items in 6 factors (Lievens et al., 2005), and the Organizational Personality Scale (OPS) which comprised 33 items in 5 factors (Slaughter et al., 2004) in order to later compare the LOCS to previous scales.

##### Demographic questionnaire

As in the pilot study, Piasentin’s (2007, Unpublished) demographic questionnaire was used in collecting detailed participant information.

#### Procedure

An online questionnaire was used in collecting data, which included all scales and measures mentioned above.

## Results

### Analysis Rationale

Given the goal of identifying the number and content of culture dimensions present in the space we judged it appropriate to use an exploratory factor analysis approach using Principle Components Analysis (PCA). For a full description and discussion of the rationale behind the use of PCA we suggest Nunnally and Bernstein (1994). As Nunnally and Bernstein (1994) note, when properly interpreted most exploratory factor analysis approaches should arrive at a similar conclusion regarding the number of factors. However, they note “We therefore suggest that an exploratory study generally use a component solution, assuming the principles of good factor analytic design have been followed…” (p. 515). The primary shortcoming of the PCA is a tendency to identify a large number of trivial factors based on the low threshold of eignevalues > 1 (Nunnally and Bernstein, 1994). As a result, researchers have typically added an additional step of using a scree plot (Cattell, 1966) to determine the cutoff point for meaningful factors rather than the less strict eigenvalues > 1 approach. Typically the scree or debris items can then be removed and the analysis run again to clarify the factor structure among the remaining items. Once the number of factors is identified it is typically useful to allow rotated factors to correlate in order to extract the most interpretable solution (Nunnally and Bernstein, 1994). There are certainly other approaches to dimension identification that are suitable including Horn’s Parallel analyses (Horn, 1965). However, given that most exploratory approaches tend to yield similar results when used correctly, we judged it appropriate to use a widely used and time-tested approach of PCA for the present study and to follow up those results with the more conservative parallel analysis to confirm the number of factors.

Subsequent to determining the factor structure of the LOCS we provide hierarchical linear regression analyses to determine whether the LOCS predicts the dependent variables of Job Satisfaction, Affective Commitment, Perceived Fit, and Job Search Behaviors. In order to provide the most stringent test for our new measure, we first entered each of the competing frameworks’ culture dimensions in a control block and then entered the LOCS dimensions into the equation (see Pedhazur, 1982). In order to show prediction of incremental variance in our dependent variables by our predictor variables (LOCS dimensions) we expect that the *R*-values for the second block in the hierarchical analysis will be significant after entering the control variables in Block 1.

### Initial Results

A PCA of the 437 descriptive adjectives was performed on the data from the 325 employed participants. Consistent with the recommendations of Kim and Ferree (1981), all variables were first standardized by subtracting the mean adjective value from each adjective score and dividing it by the standard deviation. The Kaiser-Meyer-Olkin measure of sampling adequacy was 0.77, indicating that the data was suitable for principle components analysis. In addition, Bartlett’s test of sphericity was significant [χ^2^(17391) = 40531, *p* < 0.001), which indicates that sufficient correlation between variables exists to proceed with analysis.

A total of 34 factors had eigenvalues greater than 1.00, and as such it was decided that for the sake of interpretability a closer inspection be given. The scree plot showed an obvious “elbow” at the ninth factor, a method for determining the number of factors to retain that has been widely used for nearly 50 years (Cattell, 1966) and has stood the test of time (Meyers et al., 2012). As such, it was determined that items would be forced onto an 8, 9, and 10 factor solution to test interpretability. An oblique rotation was chosen in performing this analysis, as previous research suggests that for correlated factors an oblique rotation will provide a better estimate of true factors and a better simple structure than an orthogonal rotation (Fabrigar et al., 1999). Because at this point it was unknown whether the latent factors would correlate, we chose a cautious approach in allowing the factors to correlate.

Direct oblimin and promax are the two most popular methods of oblique rotation supported by SPSS. In this case, a promax rotation was chosen due to the large dataset, which would have slowed down a direct oblimin rotation (Ivancevic and Ivancevic, 2007). Ivancevic and Ivancevik have concluded that the results from the two types of oblique rotation should be very similar.

After forcing the data into 8, 9, and 10 factors and specifying an oblique rotation, we judged that the nine factor solution provided the best description of the data. Items were then chosen for deletion if they did not load on any factor greater than 0.4, or if they had crossloadings with two or more factors above 0.4. 250 adjectives were removed from the list at this stage, and the analysis rerun with the shortened adjective list. Using three iterations of this process, an additional 50 adjectives were removed until a final list of 135 organizational descriptive adjectives remained for further interpretation. The nine factors accounted for 42.5% of the total variance among these 135 items. We named this nine factor solution the Lexical Organizational Culture Scale (LOCS).

Due to the potential for PCA to overestimate the number of factors we also conducted a parallel PCA analysis (Horn, 1965) with 1000 permutations from the raw data and using a 95% confidence level for cutoffs. This analysis also found the 9 factor solution was the best fitting model. Accordingly, we conducted the rest of our analyses based on the 9 rotated factors from the LOCS PCA analyses above.

### Organizational Culture Scale Factors

**Table 1** shows the breakdown of each of the nine factors and the items included within each dimension, as well as loadings. Once identified, reliability analysis was run for each factor by testing the internal consistency (as recommended by Carmines and Zeller, 1979). Reliability scores for all scales fell above the recommended minimum levels of 0.7 (Nunnally and Bernstein, 1994), and are shown in **Table 2** along with correlations between the dimensions.

**Table 1:** Lexical organizational culture scale dimensions, items, and loadings^a^.

Innovative		Dominant		Pace		Friendly		Prestigious	
Item	Loading	Item	Loading	Item	Loading	Item	Loading	Item	Loading
Ordinary	–0.68	Huge	0.86	Organized	0.62	Happy	0.67	High End	0.72
Typical	–0.65	Gigantic	0.85	Knowledgeable	0.61	Nice	0.65	Prestigious	0.64
Boring	–0.63	Giant	0.85	Non-productive	–0.61	Warm	0.63	Upper Class	0.64
Exciting	0.60	Large	0.84	Efficient	0.60	Friendly	0.62	High Class	0.63
Extraordinary	0.60	Large scale	0.83	Effective	0.59	Hard	–0.61	Rich	0.57
Intriguing	0.59	Big	0.83	Unfocused	–0.58	Cheerful	0.58	Sophisticated	0.57
Indistinctive	–0.59	Enormous	0.82	Consistent	0.57	Demanding	–0.58	High Level	0.55
Unique	0.59	Massive	0.80	Purposeful	0.57	Likable	0.58	Extravagant	0.55
Inspirational	0.58	Small	–0.76	Competent	0.56	Approachable	0.56	Privileged	0.50
Visionary	0.58	Little	–0.69	Orderly	0.55	Tough	–0.56	Formal	0.48
Gifted	0.58	Major	0.65	Diligent	0.55	Difficult	–0.55	Superior	0.47
Special	0.57	Global	0.53	Clean	0.51	Relaxed	–0.54	Elaborate	0.47
Generic	–0.57	Powerful	0.49	Productive	0.51	Intimidating	–0.52	Exclusive	0.44
Remarkable	0.57			Neat	0.51	Inviting	0.52	Renowned	0.41
Creative	0.56			Informed	0.51	Lenient	0.50		
Interesting	0.56			Logical	0.51	Intense	–0.50		
Common	–0.56			Important	0.51	Critical	–0.50		
Original	0.54			Professional	0.50	Flexible	0.49		
Innovative	0.53			Relevant	0.50	Polite	0.49		
Adventurous	0.53			Credible	0.49	Down to Earth	0.44		
Normal	–0.52			Careful	0.44	Cooperative	0.42		
Courageous	0.51			Healthy	0.44	Generous	0.42		
Distinctive	0.51			Non-essential	–0.43	Modest	0.42		
Predictable	–0.50			Complete	0.41	Aggressive	–0.40		
Regular	–0.47								
Imaginative	0.46								
Clever	0.46								
Dynamic	0.44								
Inventive	0.43								

**Trendy**		**Corporate social responsibility**		**Traditional**		**Diverse**	
**Item**	**Loading**	**Item**	**Loading**	**Item**	**Loading**	**Item**	**Loading**

Successful	0.644	True	0.649	Old fashioned	–0.680	Diverse	0.629
Popular	0.612	Conscious	0.643	Old school	–0.676	Discriminating	–0.591
Competitive	0.539	Trustworthy	0.625	Up to date	0.633	Multicultural	0.570
Accomplished	0.537	Unselfish	0.595	Modern	0.607	Inhumane	–0.565
Ambitious	0.536	Conscientious	0.504	Outdated	–0.601	Prejudiced	–0.555
Proud	0.529	Sustainable	0.499	Traditional	–0.599	Comprehensive	0.468
Marketable	0.523	Observant	0.496	Young	0.436	Racist	–0.466
Unsuccessful	–0.520	Valuable	0.467				
Confident	0.499						

*^*a*^N = 325.*

**Table 2:** Correlations and reliabilities among lexical organizational culture scale dimensions.

Dimension	1	2	3	4	5	6	7	8	9
(1) Innovative	(0.90)								
(2) Dominant	0.06	(0.93)							
(3) Pace	0.54^∗∗^	0.20^∗∗^	(0.88)						
(4) Friendly	0.44^∗∗^	–0.18^∗∗^	0.57^∗∗^	(0.89)					
(5) Prestigious	0.52^∗∗^	0.45^∗∗^	0.45^∗∗^	0.04	(0.84)				
(6) Trendy	0.48^∗∗^	0.41^∗∗^	0.61^∗∗^	0.36^∗∗^	0.58^∗∗^	(0.86)			
(7) Corporate social responsibility	0.57^∗∗^	0.04	0.71^∗∗^	0.58^∗∗^	0.32^∗∗^	0.45^∗∗^	(0.86)		
(8) Traditional	0.40^∗∗^	0.03	0.36^∗∗^	0.34^∗∗^	0.21^∗∗^	0.44^∗∗^	0.35^∗∗^	(0.79)	
(9) Diverse	0.36^∗∗^	0.10	0.63^∗∗^	0.62^∗∗^	0.16^∗^	0.46^∗∗^	0.54^∗∗^	0.35^∗∗^	(0.76)

*^∗∗^p < 0.01*.

### Relationships Among Outcome Measures and the Nine Organizational Culture Factors

In addition to the culture adjectives, participants completed four additional measures to assess important attitudes and behaviors that have previously been shown to relate to perceptions of corporate culture. For example, O’Reilly et al. (1991) used the OCP to predict job satisfaction and normative commitment with significant results and Lievens et al. (2005) suggested that their Symbolic Trait Dimensions could be used in predicting Person-Organization (P-O) fit. Job search behaviors are an important outcome variable of interest to organizations, given the substantial cost of employee turnover (Tziner and Birati, 1996) and warrant inclusion as a measure of interest. Furthermore, the inclusion of self-reported job search behaviors helps reduce potential common method variance associated with having only attitudinal measures.

Scale scores were calculated for each of the nine LOCS dimensions by averaging the adjective scores for each dimension (i.e., unit weighting). These scales were subsequently used in linear regression equations to predict the four outcome variables of interest. Zero order correlations are shown in **Table 3**. In general, the LOCS was able to significantly predict all four outcome measures, in support of Hypothesis 1.

**Table 3:** Predicting organizational outcomes with the lexical organizational culture scale^a^.

Dimension	Job satisfaction	Perceived fit	Job search behaviors	Affective commitment
Innovative	0.28**	0.27**	0.06	0.41**
Dominant	–0.12*	–0.13*	0.01	–0.13*
Pace	0.12	0.11	–0.13	–0.03
Friendly	0.24**	0.26**	0.07	0.13*
Prestigious	0.03	0.00	0.19*	0.02
Trendy	0.02	0.14*	–0.26**	0.05
Corporate social responsibility	0.18**	0.03	0.08	0.21**
Traditional	0.10*	0.05	0.15*	–0.05
Diverse	–0.04	–0.03	–0.21**	0.02
Overall effect				
Δ*F*(9,316)	38.86**	25.20**	4.36**	26.27**
Δ*R*^2^	0.53**	0.42**	0.11**	0.43**

*^*a*^N = 326. The values in the table are standardized beta weights. ^∗^p < 0.05, ^∗∗^p < 0.01.*

#### Job Satisfaction

The five item Brayfield–Rothe Job Satisfaction Scale used in this study (Brayfield and Rothe, 1951) demonstrated excellent reliability (α = 0.91). Overall, the nine LOCS dimensions were found to be a significant predictor of job satisfaction [*R*^2^ = 0.53, *F*(9,316) = 38.92, *p* < 0.001]. In particular, the Innovative scale was the strongest predictor of satisfaction followed by Friendly, and Corporate Social Responsibility. A Dominant image was significantly negatively associated with job satisfaction.

#### Fit Perceptions

Reliability for the subjective fit items used in this study (Piasentin and Chapman, 2007) was fair (α = 0.76). The nine LOCS dimensions were also a significant predictor of fit perceptions [*R*^2^ = 0.42, *F*(9,316) = 24.99, *p* < 0.001] overall. Innovative and Friendly were the strongest predictors, while Dominant was significantly negatively related to fit perceptions.

#### Job Search Behaviors

The reliability of Blau’s (1994) job search behaviors scale was high (α = 0.90). The nine dimensions also significantly predicted actual job search behaviors [*R*^2^ = 0.11, *F*(9,316) = 4.32, *p* < 0.001]. Individuals who viewed their organizations as Trendy and Diverse were less likely to engage in active job search behaviors, whereas individuals in Traditional and Prestigious organizations were more likely to have submitted resumes, attended interviews and engaged in other job search behaviors.

#### Affective Commitment

Allen and Meyer’s (1990) eight item affective commitment scale demonstrated good reliability (α = 0.83). The LOCS was a significant predictor of affective commitment, [*R*^2^= 0.43, *F*(9,316) = 26.27, *p* < 0.001] overall. Corporate Social Responsibility, Friendly, and Innovative were all found to have a significant positive relationship with affective commitment, while a significant negative relationship was found with the Dominant dimension.

### Comparing Previous Scales and the LOCS

In order to assess whether the LOCS provided incremental validity in predicting a variety of important organizational attitudes and behaviors, scale scores were calculated for several existing measures of organizational culture that were created using methods other than a lexical approach. Participants’ ratings of the adjectives found in the Organizational Personality Scale (OPS), Corporate Character Scale (CCS), and Symbolic Scale (SbS) were used in comparing the LOCS with previous measures of perceived corporate culture.

Scale scores were calculated for each of the dimension of CCS, OPS, and SbS by averaging the adjective scores for each dimension (i.e., unit weighting), as was done with the LOCS dimensions. These scales were subsequently used in hierarchal linear regression equations to predict the four outcome variables of interest in order to compare the models. In order to determine whether our new model of culture provided incremental prediction beyond existing culture measures, we entered each of the dimensions of the competing culture models first in the equation, followed by the dimensions from our new model. Thus, an *F* statistic could be provided that ascertains whether incremental prediction was achieved. **Tables 4–6** show that the LOCS was able to account for incremental variance above and beyond each previous measure of organizational culture.

**Table 4:** Incremental variance of the LOCS over the OPS^a^.

	Job satisfaction		Perceived fit		Job search behaviors		Affective commitment	
Predictor	Step 1	Step 2	Step 1	Step 2	Step 1	Step 2	Step 1	Step 1
**Control variables**								
Thrift	–0.15**	–0.07	–0.15**	–0.11*	0.11	0.10	–0.09	0.00
Style	0.07	0.02	0.10*	0.09	0.19**	0.08	0.07	0.02
Innovativeness	0.18**	–0.09	0.16**	0.01	0.07	0.01	0.25**	–0.08
Dominance	–0.02	0.07	–0.01	0.06	–0.21**	–0.14	–0.11*	–0.14
Boy Scout	0.49**	0.10	0.44**	0.25*	–0.06	0.12	0.42**	0.25*
**Organizational culture variables**		
Innovative		0.33**		0.24**		0.03		0.45**
Dominant		–0.12*		–0.10		0.02		–0.10
Pace		0.08		0.05		–0.10		–0.06
Friendly		0.23**		0.15		–0.05		–0.01
Prestigious		0.01		–0.07		0.15		0.02
Trendy		–0.05		0.04		–0.16		0.12
Corporate social responsibility		0.16*		–0.01		0.07		0.16*
Traditional		0.08		0.03		0.16*		–0.04
Diverse		–0.07		–0.06		–0.18*		0.01
*R*^2^	0.45**	0.54**	0.40**	0.44*	0.08**	0.13*	.37**	0.44**
Adjusted *R*^2^	0.44**	0.52**	0.39**	0.42*	0.06**	0.09*	.36**	0.42**
Δ*R*^2^	0.45**	0.08**	0.40**	0.04*	0.08**	0.05*	.37**	0.08**

*^*a*^N = 326. The values in the table are standardized beta weights. ^∗^p < 0.05, ^∗∗^p < 0.01.*

**Table 5:** Incremental variance of the LOCS over the CCS^a^.

	Job satisfaction		Perceived fit		Job search behaviors		Affective commitment	
Predictor	Step 1	Step 2	Step 1	Step 2	Step 1	Step 2	Step 1	Step 1
Control variables								
Machismo	0.01	0.02	–0.09	–0.09	0.05	0.04	0.03	–0.02
Enterprise	0.40**	0.20*	0.33**	0.11	0.05*	–0.03	0.35**	0.17
Competence	–0.10	–0.10	0.05	0.08	–0.10	0.13	–0.04	0.07
Chic	–0.09	–0.23**	–0.05	–0.15	0.17*	0.22	–0.07	–0.26**
Agreeableness	0.46**	0.20	0.29**	0.11	–0.12	–0.02	0.36**	0.12
Ruthlessness	–0.17**	–0.09	–0.14*	–0.04	–0.02	0.01	–0.14*	–0.06
Informality	–0.07	–0.05	–0.01	–0.01	0.06	0.03	–0.08	–0.03
**Organizational culture variables**	
Innovative		0.18*		0.26**		0.04		0.36**
Dominant		–0.12*		–0.14*		–0.01		–0.15**
Pace		0.08		0.07		–0.16		–0.09
Friendly		0.14		0.15		0.08		0.05
Prestigious		0.21*		0.11		–0.02		0.22*
Trendy		0.03		0.09		–0.30**		0.01
Corporate social responsibility		0.11		–0.04		0.09*		0.12
Traditional		0.05		0.01		0.16*		–0.10
Diverse		–0.07		–0.06		–0.17*		–0.02
*R*^2^	0.52**	0.55**	0.39**	0.43*	0.05*	0.13**	0.37**	0.45**
Adjusted *R*^2^	0.50**	0.53**	0.38**	0.40*	0.03*	0.08**	0.36**	0.42**
Δ*R*^2^	0.52**	0.04**	0.39**	0.04*	0.05*	0.08**	0.37**	0.08**

*^*a*^N = 326. The values in the table are standardized beta weights. ^∗^p < 0.05, ^∗∗^p < 0.01.*

**Table 6:** Incremental variance of the LOCS over the SbS^a^.

	Job satisfaction		Perceived fit		Job search behaviors		Affective commitment	
Predictor	Step 1	Step 2	Step 1	Step 2	Step 1	Step 2	Step 1	Step 2
**Control variables**								
Sincerity	0.32**	0.01	0.16*	–0.07	–0.10	–0.00	0.21**	–0.05
Ruggedness	–0.11*	0.03	–0.13*	–0.01	–0.06	–0.11	–0.06	0.04
Prestige	0.08	–0.00	0.18**	0.16	–0.26**	–0.23*	0.10	0.07
Excitement	0.18**	0.01	0.05	–0.11	0.35**	0.31**	0.18**	–0.04
Competence	–0.05	–0.03	–0.03	0.04	0.04	0.07	–0.01	0.13*
Cheerfulness	0.22**	0.02	0.31**	0.07	–0.06	0.09	0.23**	0.05
**Organizational culture variables**	
Innovative		0.25**		0.32**		–0.15		0.42**
Dominant		–0.12*		–0.15**		0.00		–0.17**
Pace		0.12		0.08		–0.10		–0.09
Friendly		0.25**		0.25*		–0.01		0.16
Prestigious		0.03		–0.05		0.22*		–0.03
Trendy		0.02		0.09		–0.23**		0.01
Corporate social responsibility		0.18**		0.03		0.10		0.20**
Traditional		0.10*		0.07		0.11		–0.03
Diverse		–0.03		–0.04		–0.20*		0.03
*R*^2^	0.45**	0.53**	0.36**	0.43**	0.09**	0.16**	0.36**	0.44**
Adjusted *R*^2^	0.44**	0.50**	0.35**	0.40**	0.07**	0.12**	0.35**	0.41**
Δ*R*^2^	0.45**	0.08**	0.36**	0.07**	0.09**	0.08**	0.36**	0.08**

*^*a*^N = 326. The values in the table are standardized beta weights. ^∗^p < 0.05, ^∗∗^p < 0.01.*

#### Job Satisfaction

The LOCS accounted for 8.4% of the variance in job satisfaction over and above variance accounted for by the OPS, 7.9% of the variance over the SbS, and 3.5% of the variance in job satisfaction incremental to the CCS. As such, Hypothesis 2 found full support.

#### Job Search Behaviors

The LOCS accounted for 5.3% of the variance in job search behavior over and above the OPS, 7.7% of the variance in job search behavior over and above the SbS, and 8% of the variance above and beyond the CCS. This incremental variance was significant in all cases, lending full support to Hypothesis 3.

#### Subjective Fit

The LOCS accounted for 3.7% of the variance in subjective fit incremental to the CCS, 3.8% of the variance incremental to the OPS, and 6.8% of the variance in subjective fit incremental to SbS, supporting Hypothesis 4.

#### Affective Commitment

The LOCS contributed a significant amount of variance in affective commitment over the SbS, with 8.2% of the variance contributed above the other scale, in addition to 7.6% of the variance over the OPS, and 8.1% of the variance over the CCS, fully supporting Hypothesis 5.

## Discussion

The main purpose of this study was to determine the dimensions of organizational culture, utilizing an inductive, lexical strategy. A principal components analysis of 437 organization descriptive adjectives revealed a nine factor solution, including the dimensions of: Innovative, Dominant, Pace, Friendly, Prestigious, Trendy, Corporate Social Responsibility, Traditional, and Diverse. Although other organizational culture type scales exist, many of these have used previous theory, including human personality theory, to guide survey construction. While this approach has been very useful, the current study frees itself from the constraints of measuring organizational culture from a pre-determined framework and instead employs an inductive lexical approach to cast the domain net significantly wider. This approach has proved to be a major contribution to the understanding of human personality in identifying two major personality models (the Five Factor Model and HEXACO models). We believe it makes a similar contribution here. The current study is unique in utilizing the lexical strategy and basing dimensions upon a large, comprehensive, organization-specific word set. Due to the larger number of adjectives employed in this study, identification of additional dimensions of subjective organizational culture that had not been included in previous scales was possible.

The second purpose of the present study was to investigate how the identified factors influence several outcome variables. In this case, the variables of interest were job satisfaction, fit perceptions, job search behaviors, and affective commitment. Regression analysis was used to determine if each of the identified dimensions was predictive of the outcomes both alone and as a full scale, and hierarchical regressions were used to determine the incremental variance contributed by the LOCS over earlier scales.

In the case of job satisfaction, five of the nine LOCS dimensions were able to significantly predict satisfaction (Corporate Social Responsibility, Friendly, Dominant, Innovative, and Traditional), which supported Hypothesis 1a. It is interesting to note that the Dominant dimension was a negative predictor, suggesting that employees working for companies that they consider dominant will have lower job satisfaction. This finding may be attributed to the presumption that large corporations are often perceived as faceless or lacking in warmth. For organizations, this finding suggests that working toward a friendly, innovative corporate image may benefit the organization by increasing employee satisfaction, an important goal as employees with higher levels of job satisfaction have been found to have lower levels of absenteeism (Cohen and Golan, 2007).

The LOCS was also found to act as a significant predictor of job search behaviors, in support of Hypothesis 1b. A closer look shows that four dimensions are responsible for this significance, with employees less likely to engage in job search behaviors when employed in a diverse, trendy organization and more likely to engage in these behaviors when working for a company perceived as prestigious and traditional. Given the young mean age of participants, it makes sense that the Traditional dimension would be related to a greater amount of job search behaviors, as a young working population may be more likely to desire to work in a “hip” organization, rather than one identified as outdated. However, the finding that participants working in organizations they considered prestigious were more likely to engage in job search behaviors is less clear.

Overall, the LOCS was found to significantly predict subjective person-organization (P-O) fit, in line with Hypothesis 1c. Prestigious, Dominant, Innovative, and Trendy were the four dimensions which significantly predicted subjective P-O fit perceptions. Once again, Dominant was found to have a negative relationship with subjective fit, and again it could be hypothesized that the participants in this sample felt that they were unable to make a connection with large organizations, lowering perceptions of fit. For organizations, boosting a friendly, innovative, trendy work environment could increase levels of subjective fit, thereby increasing many other outcomes which have been found to be affected by levels of subjective fit, including turnover, work attitudes, and work performance (Dawis and Lofquist, 1984; Schneider, 1987; Tziner, 1987).

Finally, the LOCS was able to significantly predict affective commitment, in support of Hypothesis 1d. The dimensions most closely positively associated with affective commitment are Corporate Social Responsibility, Friendly, and Innovative. Given the meaning behind affective commitment, these relationships make sense. In addition, the Dominance dimension was negatively related to affective commitment, possibly for the same reasons presented in the case of Hypotheses 1a and 1c. The prediction and influence of affective commitment should also be a key goal for organizations, as previous research has shown that affective commitment is negatively related to withdrawal cognition and turnover, as well as positively related to organization relevant outcomes such as attendance, performance, organizational citizenship behaviors (Meyer et al., 2002).

The LOCS accounted for variance over and above OPS, CCS, and the SbS in predicting job satisfaction, in support of Hypothesis 2. Likewise, strong evidence was found for the LOCS in predicting incremental variance above all three other measures of subjective organizational culture for the other three outcome variables. As such, Hypotheses 3, 4, and 5 were all fully supported. As a whole, this set of findings provides strong support for the use of the LOCS in future research as a more comprehensive scale of organizational culture.

There is some overlap between the LOCS and the dimensions included in previous instruments such as the OPS, CCS, and SbS, but the scales are different in several important ways. The LOCS was created using a lexical approach, with no prior expectations. As such, the list of adjectives used in the LOCS is more comprehensive than any of the three other measures, allowing for more specificity. In addition, some dimensions of the LOCS were identified that were fully unique. The Diverse dimension, as an example, correlates with several other dimensions in previous scales, but is unique in the items of which it is composed. Additionally, the Dominant scale did not correlate very strongly with any dimension of any other scale, suggesting that this important dimension is unique to the LOCS.

Another major difference between the LOCS and previous scales lies in the LOCS’ addition of both positively and negatively valenced words within each dimension. For example, under the Friendly dimension, words like “likable” had a strong positive loading on the factor, whereas words like “intimidating” loaded negatively. In previous scales this dichotomy was not allowed. For instance, the CCS includes two related but opposite dimensions; Agreeableness and Ruthlessness. In the LOCS these two factors are subsumed under the Friendly dimension, in which words both strongly related to and strongly contrary to the dimension are loaded.

The initial word list used in this experiment was organization specific and did not come from other sources, such as brand and human personality literature, allowing for a more comprehensive, organization-specific measure, a unique strength of this methodology in comparison to previous related studies. It is also worth noting that this methodology also allowed us to survey a large number of organizations in diverse industries, whereas other studies of organizational culture perceptions used specific organizations (e.g., large well known organizations). This adds to the generalizability of the findings and allows the conclusion to be drawn that the results found are representative of a wide range of jobs in a wide range of industries. It is precisely due to these strengths that the scale is generic in the sense that it is a comprehensive measure of the important dimensions upon which individuals evaluate similarities and differences between organizations.

There are numerous practical implications of the LOCS. The scale has value as a diagnostic tool by identifying aspects of organizational culture. Once an organization is able to evaluate how their organizational culture is perceived by employees, applicants, or other groups of interest, an organization can work on altering or improving their image on certain specific dimensions. For example, organizations can compare how customers and employees view the company and take action to align their current culture with their desired image for the target group. Similarly, an audit of this sort could be useful in creating a positive image for potential applicants, once it is identified which dimensions have the most importance to this group.

In order to attract the highest quality applicants in the early stages of recruitment, organizations must differentiate themselves from their competitors. Research has shown that organizational image perceptions influence applicant attraction and job pursuit intentions (Gatewood et al., 1993; Highhouse et al., 2003; Lemmink et al., 2003; Slaughter et al., 2004). As such, organizations must ensure that applicants are familiar with their corporate images, and that those images are sending positive signals about the organization. One way that organizational image perceptions influence intentions is through an applicant’s inference of job attributes and anticipated feelings of pride related to working for a particular organization (Gatewood et al., 1993). Collins (2007, p. 181) argued that “because job seekers’ employer knowledge affects application behavior, it is critical for recruiters to understand how to systematically influence these beliefs.” Fombrun and Shanley (1990) suggested that organizations that have a positive image attract better job applicants. Conversely, this could mean that if an organization elicits a negative image in the minds of prospective employees, many potential applicants will be unwilling to enter the applicant pool- especially those applicants who are most highly sought after, as the highest qualified applicants will likely have the greatest number of choices and therefore be more discriminating in job choice (Murphy, 1986). If organizations are able to accurately assess the dimensions of their image this would enable them to tailor their recruitment messages to increase the applicant response rate (Rynes, 1991).

A potential criticism of having current employees evaluate the company in which they work to determine organizational image is that is that the data may reflect personal experiences with the company or relationships with other employees. Organizational culture denotes all possible perspectives of an organization by virtue. Although perceived culture of a company held by a current job incumbent may differ from an outsider’s view, the same dimensions are relevant and reflective of the versatility inherent in this measure, and as such we believe that the LOCS is a useful tool for measuring and defining the different organizational images held by different stakeholders. It would be beneficial for future researchers to investigate similarities and differences between organizations in the same industry.

While the methodology employed in this study has very strong benefits, the lexical approach may also be criticized as being atheoretical. However, the study of organizational culture lends itself well to a lexical approach. The results of this study have shown that using a deductive approach to identifying culture dimensions, as the previous related studies have, missed several key aspects of culture.

In addition, it should be noted that several of the LOCS dimensions are very highly correlated, with the possibility of multicollinearity between factors. In order to get the best simple structure for the factors, Fabrigar et al. (1999) recommendation to analyze the data using an oblique rotation was used. This allowed the nine culture dimensions to correlate, a necessity in this type of research where it would be unlikely that all dimensions would be orthogonal. Despite these steps, certain factors, such as Corporate Social Responsibility and Pace continue to have very high correlations. Future research may wish to collapse highly related factors to prevent the issues that come with multicollinearity.

One limitation of this research is the composition of the sample in respect to the outcome variables we investigated. The participants were all university students and the majority were employed part-time. With this is mind, it could be hypothesized that in a different population other dimensions of the LOCS may be found to have a stronger or weaker relationship with certain outcome variables. For example, in this student sample the Traditional dimension was found to relate to greater job search behaviors. In a population of older workers it may be that the reverse could be found. In addition, it could be hypothesized that in an applicant sample certain factors, such as Friendly, may be more salient than in this sample, as friendly behaviors have been shown in previous research to have an impact on applicant attraction (Goltz and Giannantonio, 1995). Lastly, our sample consisted of an overrepresentation of women relative to the general population. This has the potential to influence the perceptions of the organizations through a number of potential mechanisms such as gender-based biases in perceptions of culture or self-selection into industries and organizations based on gender. Future research should examine the robustness of the factor structure in a more gender balanced sample.

### Future Research

Future research that focuses on full time employees would be beneficial. The sample used in this study was primarily comprised of part time workers, who could have brought different attitudes and perceptions of their organizations than older, full time workers. Independent samples *t*-tests were carried out to determine if there were differences between the groups. Full time workers were found to be older [*t*(323) = 3.65, *p* = -0.002]. A marginally significant difference was also found in ratings on the Traditional dimension between part time workers and full time workers [*t*(323) = -1.89, *p* = 0.06], which supports the theory that younger workers are less inclined to perceive tradition as favorable in an organization. However, no differences between groups were reported for the four outcome variables.

Future research that investigates how an individual’s personality interacts with these outcome variables and the nine dimensions of organizational culture offers the possibility of providing additional insight into employee attraction. For example, it could be hypothesized that individuals who score high on the personality dimension of Agreeableness would find the Friendly dimension more important than the others, or that an individual rated highly on honesty-humility would find the Corporate Social Responsibility dimension more important in an employer.

Another important question raised by these results concerns the degree a factor can positively or negatively impact organizational outcomes in conjunction with other dimensions. For example, the results found in this study suggest that a highly dominant organization is more likely to have employees with lower job satisfaction. However, friendliness and innovation have been found to positively relate with satisfaction. It would be worthwhile to examine how employees react to an interaction of these dimensions.

More outcome variables are also needed to continue testing this scale. While we have provided what we believe are four of the most important outcome variables for organizations, relating the LOCS to other outcomes, such as counterproductive workplace behaviors and actual turnover would add to the usefulness in a wider range of settings.

Finally, the LOCS needs to be validated outside of a North American context. Other lexical studies, such as the Big Five, have found support in other countries (McCrae et al., 1998). Given the growing international business environment, it is important to determine whether this scale can be generalized to organizational culture in other countries.

## Ethics Statement

This study was carried out in accordance with the recommendations of the University of Calgary Research Ethics Board with written informed consent from all subjects in accordance with the Declaration of Helsinki. The protocol was approved by the University of Calgary Research Ethics Board.

## Author Contributions

DC conceived of this research project and supervised the student contributions. He was responsible for the research design, analysis, and overall writing duties as well as handling all submission and revision tasks. PR was responsible for data collection and identified the adjectives used in the study under the supervision of DC. MC updated the manuscript and provided editorial assistance. She also updated analyses where necessary.

## Conflict of Interest Statement

The authors declare that the research was conducted in the absence of any commercial or financial relationships that could be construed as a potential conflict of interest. The reviewer CG-A and handling Editor declared their shared affiliation.
